# The Impact of Environmental and Genetic Factors on Bone Quality in Monozygotic and Dizygotic Twins

**DOI:** 10.3390/biomedicines10102360

**Published:** 2022-09-22

**Authors:** Elżbieta Tabor, Małgorzata Bach, Aleksandra Werner, Bogna Drozdzowska, Wojciech Pluskiewicz

**Affiliations:** 1 Department and Clinic of Internal Diseases, Diabetology, and Nephrology, Faculty of Medical Sciences in Zabrze, Medical University of Silesia, 40-055 Katowice, Poland; 2 Department of Applied Informatics, Silesian University of Technology, 44-100 Gliwice, Poland; 3 Department of Pathomorphology, Faculty of Medical Sciences in Zabrze, Medical University of Silesia, 40-055 Katowice, Poland; 4 Metabolic Bone Diseases Unit, Department and Clinic of Internal Diseases, Diabetology, and Nephrology, Faculty of Medical Sciences in Zabrze, Medical University of Silesia, 40-055 Katowice, Poland

**Keywords:** twins, monozygotic, dizygotic, bone, quantitative ultrasound

## Abstract

The purpose of the research was to assess the genetic and environmental influences on bone properties. One hundred thirty-two pairs of twins (99/33 monozygotic/dizygotic) underwent anthropometric measurements and phalangeal quantitative ultrasound (DBM Sonic 1200, Igea, Italy) measuring the amplitude speed of sound (AD-SoS, m/s). The mean age was 16.78 ± 12.35 years for monozygotic twins and 14.30 ± 8 years for dizygotic. Interpair and intrapair correlations between twins were calculated. In the groups of monozygotic and dizygotic twins, Ad-SoS correlated significantly with age (r = 0.56–0.73, *p* < 0.05), weight (r = 0.73–0.78, *p* < 0.05), and height (r = 0.80–0.81, *p* < 0.05). The strongest intrapair correlation (r = 0.99–0.998) was noted in monozygotic females for Ad-SoS, weight, and height. There was a statistically significant correlation between the intrapair difference of Ad-SoS and age but only in the groups of monozygotic and dizygotic females (r = 0.281, r^2^ = 0.079, and *p* = 0.028; r = 0.544, r^2^ = 0.296, and *p* = 0.01, respectively). After age adjustment, it was estimated that 28.62% of Ad-SoS in women and 13.2% of Ad-SoS in men was explained by genetic influence, leading to the conclusion that Ad-SoS changed with age, weight, and height. The strongest correlation between pairs of twins was observed in monozygotic twins. The differences in bone values between female twins arose with age, which indicated the role of environmental factors.

## 1. Introduction

Bone tissue is essential for humans not only because of its scaffolding and protective role for inner organs but also due to the bone marrow’s environment and calcium storage. Over their lifetime, bones are continuously remodelled. This process is lifelong and is controlled at many levels. Any imbalance in either of them leads to excessive bone resorption or overbuilding. Starting from birth, this process is dominated by bone building until peak bone mass is reached during one’s 20s. From this point, bone modelling is overbalanced by bone resorption. Its rate is dependent on many factors. Some of them are nonmodifiable such as age, gender, ethnicity, genetic factors, and others that depend on lifestyle and comorbidities including dietary habits, alcohol abuse, cigarette smoking, physical activity, chronic use of proton pump inhibitors, glucocorticoids or anticonvulsants, chronic kidney disease, and imbalanced thyroid gland disease [1,2]. In the foetal stage and early after birth, genes are assumed to have a major influence on bone modelling, while environmental factors modify this process during one’s lifetime. Monozygotic twins share the same genes (opposite to fraternal twins), but very often they differ from each other in adulthood in terms of bone health [3,4].

According to guidelines by the World Health Organization (WHO), the gold standard for bone parameter assessment is dual-energy X-ray absorptiometry (DXA). This method is regarded as safe, but its use is still limited due to the fact of its use of ionizing radiation. The main information that DXA provides is bone density, but it does not allow for the assessment of its structural and qualitative properties [5]. Moreover, the costs for a densitometer and its maintenance are relatively high. A good alternative to this technique is quantitative ultrasound, as its devices are free of radiation, small, portable, and cheap. Additional advantages of using QUS are the possible quantitative and qualitative insights into bone elasticity and microstructure. More importantly, QUS can discriminate between healthy individuals and osteoporotic patients [6,7,8].

The aim of the present study was to assess, with use of phalangeal QUS, whether genes are the main directors of bone modelling in early years and whether their role is substituted by environmental impacts on the growth process of a cohort of twins.

## 2. Materials and Methods

Volunteers for this research were recruited from participants of the International Twin Festival in Szczecin (Poland), who came to the festival from around the whole country and participated in the research. The interview and measurements were performed at the University of Szczecin in August 2003. The research was approved by the Medical University of Silesia Bioethical Committee (approval Code: NN-013-117/03, approval date: 7 May 2003). All participants signed informed consent (in the case of children, consent was obtained from their parents). Next, any medical history of chronic diseases and medications used was collected to exclude those with secondarily impaired bone metabolism (participants with chronic renal or liver disease, thyroid hormone imbalance, intestinal surgery in the past, oncological diseases, prolonged glucocorticoids use, proton pump inhibitors, anticonvulsants, anticoagulants, loop diuretics, etc.). A detailed questionnaire is included below the text of the manuscript (see Addendum). Women who had reached menopause were also excluded. The final study group consisted of 132 pairs of twins (i.e., 264 participants: 166 females and 98 males). Among the study group, 198 of them were monozygotic (MZ) twins and 66 dizygotic (DZ). The mean age was 16.16 ± 11.44 (minimum = 4, maximum = 66). None of the participants had a history of bone fracture. Subjects younger than 12 years old were considered children.

Twins were classified as MZ or DZ according to their self-reports, assuming their accuracy up to 5% based on previous investigations [9]. Actual body weight and height were measured with the use of standard anthropometric methods. The mean weight was 45.62 ± 19.69 kg (minimum = 16, maximum = 106). The mean actual body height was 1.5 ± 0.2 m (minimum = 1, maximum = 1.87). The abovementioned characteristics for the MZ and DZ twins are presented in Table 1.

None of the participants were born with a birth weight less than 1 kg, and in 4 cases the birth weight did not exceed 1.5 kg.

All participants underwent phalangeal QUS with the use of a DBM Sonic 1200 (IGEA, Carpi, Italy) device. The probes were adjusted to the distal metaphyses of the proximal phalanges of the 2nd to 5th finger of the nondominant hand to measure the amplitude-dependent speed of sound (Ad-SoS, (m/s)). All measurements were conducted by one experienced person (W.P.). The coefficient of variation (CV%) for AD-SoS was 0.64%.

### Statistical Analysis

All statistical calculations were performed with the use of Statistica 13.3 software (StatSoft, Tulsa, OK, USA), PQStat v.1.8.2.226 (PQStat Software, Plewiska, Poland; https://pqstat.pl, accessed on 20 July 2022), and R Statistical Software v.4.0.3 (© The R Foundation; https://www.r-project.org, accessed on 20 July 2022). A *p*-value at a level of 0.05 was regarded as statistically significant.

The analysis of the twin data started with descriptive statistics that were performed on the entire dataset and with the identification of data distribution using histograms and the Shapiro–Wilk test. Descriptive data are presented as the mean value and standard deviation and as the median value with the minimal and maximal range. Then, the study population was grouped according to the twin type. The subgroups of monozygotic and dizygotic twins were analysed separately, additionally taking into account the sex of the twins (males/females) and the order of birth (A—the first-born twin; B—the second-born twin). This allowed for performing both interpair and intrapair analyses. To compare the MZ and DZ groups of twins, the correlation coefficients were observed between the following variables: sex, age, weight, height, and the value of the Ad-SoS measurements. Correlation analysis was performed using Spearman’s correlation coefficient (denoted by ρ or r_s_) between two continuous variables and the point biserial coefficient (r_pb_) between continuous and discrete variables.

Since a normal distribution for the analysed variables was not confirmed by the Shapiro–Wilk test, the Mann–Whitney U test was applied to determine whether there were differences between the medians of the studied traits in the two subgroups MZ and DZ. Additionally, intrapair analyses were performed for MZ and DZ twins separately for both genders. To test whether the abovementioned traits, such as age, weight, or height, could influence absolute intrapair differences in Ad-SoS values, multiple linear regression was also used.

The last step in the research focused on the issue of Ad-SoS heritability. To estimate the influence of the genetic and environmental components on the total phenotypic variance, the univariate twin analysis was conducted with the use of the Cholesky ACE (additive genetic, common environmental, and nonshared environmental influences) model. In this approach, genetic and environmental effects are modelled as latent (unmeasured) variables such as A, C or D, and E. A represents additive genetic factor, C stands for common (shared) environmental, D for nonadditive genetic, and E for nonshared environmental effects.

To estimate the influence of these factors on the given phenotype, several models were built, and the baseline model, which referred to the entire cohort, was compared with the models concerning sex differences. We also investigated whether genetic and environmental factors changed when adjusted for age. The mentioned adjustment was performed by including an age covariate in the model. The procedure that built and ran the Cholesky ACE twin model handled the covariate, i.e., the age variable that was intermediate in a causal sequence between the factors and the phenotype by modelling it in the means [10].

Moreover, each full ACE model that included all the latent factors was modified by gradual removal of particular components. This resulted in the creation of ACE, ADE, AE, CE, and E-only models, which enabled the ability to choose the most parsimonious model selected based on the nonsignificant χ^2^ goodness of fit statistic and the minimum value of the Akaike information criterion. In addition, the following indexes of fit were analysed: normalized chi-squared, comparative fit index (CFI), Tucker–Lewis index (TLI), incremental fit index (IFI), and root mean square error of approximation (RMSEA) with a 90% confidence interval (CI).

For the observed Ad-SoS, the best-fitting model was always the ACE model. Therefore, the dominant genetic (D) factor was not considered in the results described in the subsequent sections.

## 3. Results

The Ad-SoS values in terms of zygosity and gender are presented in Table 2.

In the groups of MZ and DZ twins, Ad-SoS correlated significantly with age (r = 0.56 and r = 0.73, respectively, and *p* < 0.05), actual weight (r = 0.73 and r = 0.78, respectively, and *p* < 0.05), and height (r = 0.81 and r = 0.8, respectively, and *p* < 0.05). There were no significant differences in Ad-SoS, weight, or height between the MZ and DZ twins.

In the intrapair analysis, there was a significant strong correlation for Ad-SoS, actual weight, and height (*p* < 0.000001) as presented in Table 3.

The strongest correlations were noted in MZ females for Ad-SoS, weight, and height, and the weakest (but still highly significant) was for DZ twins.

**Figure 1:**
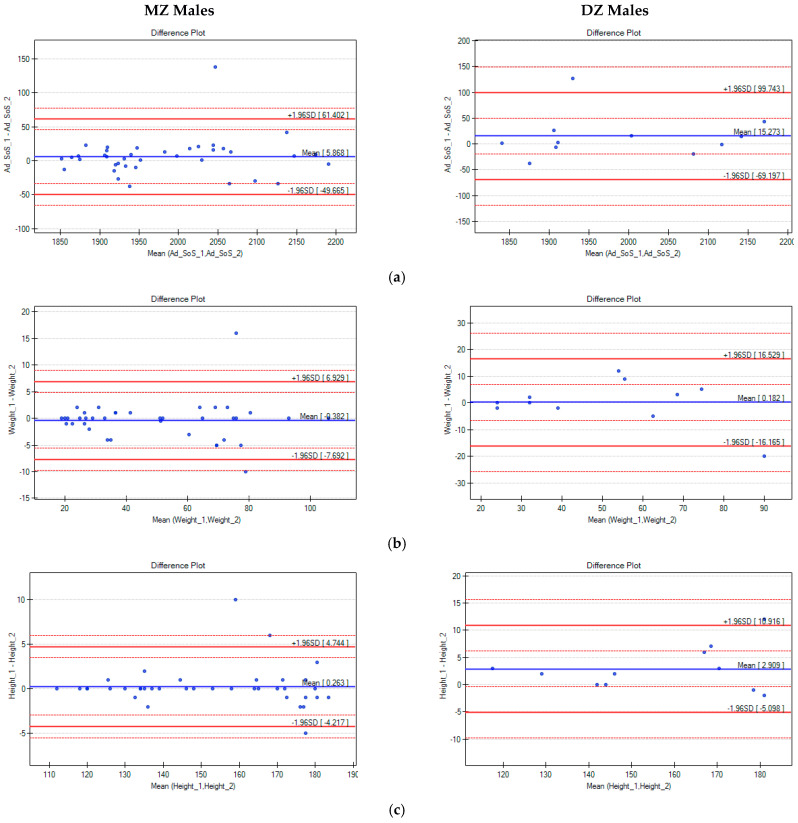
Bland–Altman plots for Ad-SoS (**a**), weight (**b**), and height (**c**) differences in pairs of male twins (the x-axis represents the mean values of the parameters in twin pairs, and the y-axis represents the intrapair differences between twin A and twin B).

**Figure 2:**
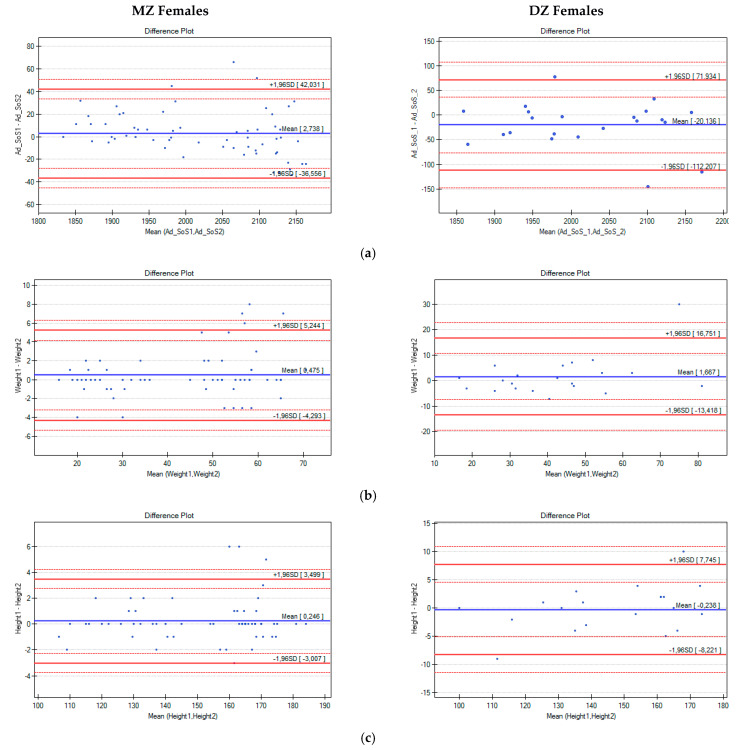
Bland–Altman plots for Ad-SoS (**a**), weight (**b**), and height (**c**) differences in pairs of female twins (the x-axis represents the mean values of the parameters in twin pairs, and the y-axis represents the intrapair differences between Twin A and Twin B).

Next, intrapair differences in Ad-SoS, weight, and height were analysed with the use of Bland–Altman plots (Figure 1 and Figure 2).

With the use of multiple linear regression, changes in the intrapair differences in Ad-SoS (ΔAd-SoS) were analysed in terms of the age, weight, and height of the twins. Both the differences in weight and height, as well as the mean values of weight and height, for twins in pairs were analysed. In all analysed cases, positive correlations were noted, which means that with the increase in weight, height, or age, the ΔAd-SoS also increased. However, only correlations between the ΔAd-SoS and age in the groups of MZ (r = 0.28, r^2^ = 0.079, and *p* = 0.025) and DZ females (r = 0.544, r^2^ = 0.296, and *p* = 0.01) and correlations of weight with the ΔAd-SoS for the DZ females (r = 0.610, r^2^ = 0.372, and *p* = 0.003 for the Δweight; r = 0.555, r^2^ = 0.308, and *p* = 0.009 for the mean weight) were statistically significant.

The Cholesky ACE model was applied to estimate the influence of genetic and environmental components on the total phenotypic variance of Ad-SoS. For the baseline model, which was built for the entire twin cohort, the standardized additive genetic parameter was 0.415. This meant that the proportion of the total variance of Ad-SoS explained by the genetic parameter variance was σ^2^_A_ = 0.415^2^ = 0.172 (17.22%). Common (C) environmental factors shared by twins reared and living in the same environment and nonshared (E) environmental factors were responsible for 79.92% and 2.76% of the variation in Ad-SoS, respectively.

The baseline model was compared with the results obtained for gender-specific models, which confirmed the differences in the influence of the genetic factor on Ad-SoS depending on sex. The heritability estimates for women were higher than for men and amounted to σ^2^_A_ = 0.467^2^ (21.8%), while for men it equalled σ^2^_A_ = 0.329^2^ (10.82%). In this case, for women 76.21% and 2.07% and for men 85% and 4.1% of the variance of Ad-SoS was attributable to C and E environmental factors, respectively.

Domain experts show that age can change the factors of the Cholesky decomposition. For this reason, the models mentioned above were adjusted for age and re-examined.

After including age as a covariate in the ACE baseline model, the estimate of the genetic additive parameter increased to 0.466. This meant that 21.7% of the total phenotypic variance for Ad-SoS was associated with genetic factors and 74.8% and 3.5% with environmental factors C and E, respectively.

The age-adjusted model created for the female twins showed that the A, C, and E factors explained 28.62%, 68.56%, and 2.72% of Ad-SoS variance, respectively. For male twins, it was 13.2%, 81.7%, and 5.1%, respectively.

In conclusion, adjusting for age increased the estimate of the heritability indices for Ad-SoS and, thus, decreased the overall impact of common (C) and nonshared (E) environmental factors. However, a more detailed analysis showed that factor C decreased, while factor E increased in age-adjusted models. This seems logical, because as twins become older, they begin independent lives, often in different environments.

An analysis of the evaluation measures, including Akaike information criterion and nonsignificant χ^2^ goodness-of-fit statistics, showed that the age-adjusted models were better fitted to the observed data.

## 4. Discussion

The most important finding given by the current study was the limited impact of genetics on bone status expressed by hand QUS measurements. Clearly, bone status was mainly influenced by environmental factors. The study’s results indicate the necessary direction of human activities in order to slow the skeletal changes with aging. We consider that the results of the current study indicate the role of environmental factors in modifying skeletal status with aging and may be helpful for practitioners in daily practice. Factors such as diet, physical activity, effective therapy of some diseases known to affect bone status, and avoidance of overtreatment with some medications are crucial for maintaining skeletal health in good condition.

Bone modelling starts in prenatal life, and approximately 80% of the mineralization process occurs in the third trimester [11,12,13]. Many factors can influence bone development such as a maternal history of cigarette smoking, alcohol abuse, comorbidities, exposure to X-ray during pregnancy, trauma, or premature rupture of membranes [14]. The strongest risk factor for bone disorders in the neonatal age are premature birth and a low birth weight. In the study group, none of the participants were born with a weight less than 1 kg (a strong risk factor for metabolic bone disease [15]). Considering the same intrauterine environment and gestational age between twin pairs, we should expect the same bone mineralization. As the study revealed, monozygotic twins had more comparable bone properties than dizygotic pairs. In a study by Howard et al. [16], similar observations were found but in a female group. The research was conducted on adult twins (both MZ and DZ) in the age range 20–83 (mean age = 53 ± 13), and one of the authors’ aims was an assessment of the role of genetic factors in bone determination. It was found that as many as 80% of QUS variance was attributable to genetic factors. This observation was confirmed by other epidemiological studies with a variance of genetic influence of 65–92% [17,18,19]. The observation of a genetic influence is consistent with other studies with the use of QUS also on different body sites [20,21]. An interesting idea was presented by Curtis et al. [22], which explains the differences in bone status in two genetically identical siblings as a result of epigenetic processes that have environmental influences on genes expression. The examples may be the effect of maternal serum vitamin D on foetal bone formation or methylation of the osteoprotegerin and RANKL genes. In the present study, the heritability estimates for Ad-SoS were not high. The additive genetic effects in the age-adjusted models accounted for just over 13.2% and 28.62% of the variance of Ad-SoS in the male and female twins, respectively. However, these results are in line with what we expected after analysing the intrapair correlations for Ad-SoS (Table 3), which were high both in the MZ and DZ twin pairs (only slightly higher in MZ). To analyse the genetic and environmental influences on the variation of Ad-SoS, we used classic twin modelling based on the fact that MZ twins share the same gene sequence (100% of their genes), whereas DZ twins share, on average, 50% of their genes. Considering this fact, relatively small proportions of Ad-SoS variation explained by additive genetic effects were consistent with the relatively small differences in the values of the intrapair correlation in MZ and DZ twins. The disagreement between our results and cited research may result from the different measurement techniques and locations as well as from the different age range of our group in comparison to the age studied by other authors. The present study group consisted of women in the premenopausal age only, which excluded possible additive hormonal effects on bone metabolism and should be considered as a strength of the study.

The process of human skeleton maturation is modulated by various factors. When it comes to QUS parameters, females usually have higher values of phalangeal Ad-SoS than males. This can easily be explained by the fact that phalanges are mostly trabecular bones that are highly sensitive to hormonal and osteometabolic changes [23,24]. In women, activation of the hypothalamic–pituitary–adrenal axis occurs earlier than in men [25]. Oestrogens are the key hormones in the regulation of bone maturity for both sexes. They are also the main determinants of epiphyseal growth, epiphyseal plate closure, and inhibition of bone resorption [26]. Available observations suggest that age-related hypothalamic–pituitary–gonadal axis hormones are sex dependent as well [27]. The main landmark in the natural history of the bone resorption rate in females is menopause. Although the oldest female participant in this study was only 60 years old, and the mean age of the study group was 17 years old, the gender-specific differences were already noticeable. No statistically significant age-dependent differences between male twin pairs in bone parameters were observed. Chwałczyńska et al. [28] and Bolanowski et al. [29] confirmed that physical fitness and muscle strength were important factors influencing Ad-SoS values in adolescents; nevertheless, the impact was seen only in older boys and in all girls. This may confirm the hypothesis that due to the fact of an earlier pubertal age, females are increasingly susceptible, as well as at an earlier age, to bone remodelling changes.

Bone quality changes significantly with age, which suggests the impact of the environment. In our study, the environmental influence after age adjustment was as high as 74.82% for common environmental factor and was stronger for men. A significant increase in intrapair differences in MZ twins was also observed in Guglielmi et al. [30,31] and Drozdzowska et al. [32]. In a study on an Italian group of MZ twins [30], a difference in bone parameters also changed significantly in a female group over 40 years old. This confirms our theory concerning hormonal influence, especially on puberty and menopause onset. In a Polish study [32], the authors described the strong impact of age on intrapair differences, both in the female and male groups. In our research, there was no statistically significant correlation of ΔAd-SoS with any of the analysed anthropometric measurements in men. However, it is worth mentioning that ΔAd-SoS increased with age in this group, but this increase cannot be considered statistically significant. This may have been caused by the relatively small group of men in the study group. Another reason may be the younger age of the male participants than in the female group. Inter-sex differences in terms genetic influence on bone mineralization were analysed by Naganathan et al. [33] on a group of MZ and DZ Australian twins with use of DXA and calcaneal QUS. An additional group included in the analysis were opposite-sex twin pairs. The results of the Australian research [33] showed no gender differences for lumbar and femoral DXA as well as calcaneal QUS. The lowest correlations of bone parameters found by the authors were in the forearms of opposite-sex twin pairs, and they concluded that there might be some gender-specific genes responsible for mineralization of different bone structures. This observation is consistent with that cited by Guglielmi et al. [30]. The distal forearm is mostly trabecular bone, whereas the distal ends of the first phalangeal diaphysis contain both cortical and trabecular bone [24], which makes this body site more representative of general skeletal status.

Extensive research on groups of twins has tried to find the answer to the question of what determines the overall fracture risk Even though there are multiple studies, they all suffer from the disadvantage that various techniques for bone assessment were used, or the groups were too small. In this study, the authors decided to use QUS for trabecular bone examination in phalanges. In this way, the examination was free of radiation, and due to the fact of its portable size, it allowed for better contact with participants and, therefore, larger group collection. The results of the phalangeal QUS reflect tenuous changes in trabecular structure and are appropriate for adolescents and adults (the mean age of the study group was 17 years old) [23,25].

Additional valuable information on the potential of QUS to follow genetics on bone status is presented by Drozdzowska and Pluskiewicz [34]. Calcaneal QUS measurements were able to predict future QUS results in the daughters of women with past fractures, and the heritability of ultrasound variables ranged between 52 and 76%. These results indicate that QUS may be helpful in daily practice and in identifying subjects with enhanced fracture risk. Especially important is the possibility of predicting future QUS results before the occurrence of the first fracture. Therefore, adequate management may be initiated before the occurrence of the first fracture.

One of the study’s limitations was the small size of the study group, especially for male twins. In males, the age range was different in MZ and DZ, which might have influenced the obtained results. The other limitation was the lack of insight into participants’ full medical history.

The study’s strength was the fact that all measurements (i.e., QUS and anthropological) were conducted by one experienced person. Moreover, the research presents calculations for both genders. Little is known regarding bone changes in men; therefore, we recommend verifying the presented results in a larger group.

In conclusion, amplitude-dependent speed of sound changes with age, weight, and height. After age adjustment, it was estimated that 28.62% of Ad-SoS in women and 13.2% of Ad-SoS in men was explained by genetic influence. The strongest correlation between pairs of twins was observed in monozygotic twins. Differences in bone parameters between female twins arose with age, which indicates the role of environmental factors. In men, the impact of environmental factors was stronger than in women. Nevertheless, this conclusion requires confirmation in a larger group of male twins.

Addendum:

Questionnaire;

- Sex;
- Age in years;
- Weight in kg;
- Height in meters;
- Menarche (year);
- Menopause (year);
- Medications used;
- Undelaying diseases;
- Prior fracture: yes/no.

## Figures and Tables

**Table 1:** Demographic characteristics.

	Mean	Minimum	Maximum	SD
	**MZ Females—122 Participants**			
Age	15.95	4	47	9.55
Weight (kg)	43.14	16	71	16.17
Height (m)	1.52	1.06	1.84	0.2
	**MZ Males—76 Participants**			
Age	18.1	4	66	15.8
Weight (kg)	49.9	19	106	24.25
Height (m)	1.53	1.12	1.84	0.2
	**DZ Females—44 Participants**			
Age	14.5	4	44	8.86
Weight (kg)	42.5	16	90	17.3
Height (m)	1.47	1	1.75	0.2
	**DZ Males—22 Participants**			
Age	14	6	29	6.2
Weight (kg)	50.5	23	100	21.9
Height (m)	1.57	1.16	1.87	0.2

**Table 2:** Mean Ad-SoS for gender and zygosity classification.

	Number of Twins	Mean Ad-SoS (m/s)	SD	Minimum	Maximum
MZ males	76	1982.91	95.13	1848	2193
MZ females	122	2023.91	100.21	1833	2175
DZ males	22	1989.55	116.9	1840	2192
DZ females	44	2018.52	96.7	1835	2229

**Table 3:** Intrapair correlations for the MZ and DZ twins.

Parameter	MZ Males	MZ Females	DZ Males	DZ Females
Ad-SoS	0.978	0.990	0.966	0.938
Weight	0.994	0.994	0.964	0.949
Height	0.997	0.998	0.991	0.991

## Data Availability

From the corresponding author.
